# Philadelphia Hospital

**Published:** 1839-02-02

**Authors:** 


### CLINICAL LECTURE.

### PHILADELPHIA HOSPITAL.

ON PNEUMONIA.

*Saturday, Jan. 5th.*—Dr. GERHARD remarked—Gentlemen: You will find no subject more interesting than pneumonia, and you will meet with few that offer greater difficulties. I mean, that the disease assumes so many different forms, and presents so many complications, that it gives rise on many occasions to great embarrassment, and often baffles completely the tact of the most careful observers. In its simple form, when it occurs as idiopathic pneumonia, no disease is more readily recognised; but when the affection assumes a character which is somewhat anomalous, the ordinary signs of the disease are in a great measure concealed, and the symptoms which indicate lesions of other organs complicating the original disease, become more prominent than those of the lungs.

You will not, however, be interested in the study of pneumonia only because the disease is at times of difficult prognosis, but because its complications have a most important influence upon its ratio of mortality. It is comparatively free from danger when it occurs in adults who do not labour under any unusual state of ill health; but it becomes of great gravity, and is often mortal, in the young, the aged, and in those who are enfeebled by previous disease. Hence pneumonia is always a disease which should excite the most lively solicitude when it occurs in individuals placed under these unfavourable circumstances.

Besides the unfavourable influence of those causes which enfeeble the strength of the patient, you will find that pneumonia, when it occurs in patients of a robust constitution, and enjoying a full share of health, may present unusual severity of symptoms, and prove extremely mortal. This arises, in most cases, from the character of the disease, as influenced by the epidemic tendency of the year in which it occurs; hence, in our inquiries into the history of the disease, we shall be obliged to compare the cases which occur in one year with those previously observed, or we should be led towards very erroneous conclusions. In one year pneumonia may be frequent, but the cases may be of the simple inflammatory kind, and remarkably exempt from complications. Such cases yield an insignificant proportion of fatal cases. In another year, you may find the whole character of the disease, as it were, changed. The simple inflammatory, or sthenic cases become rare, while those which are termed asthenic, and sometimes differ but little from gangrene of the lungs, become much more frequent; in a third year, the disease may be characterized by the extreme violence of the cerebral or abdominal complications.

Within the last five years, I have seen illustrations of all these varieties of the disease. Thus, in the winter of 1834–5, the disease was sthenic, and very free from complications. In the next year, it was less prevalent, and many cases were marked by great prostration, and the mortality was proportionably greater than it had been in the previous year. At the end of the winter, typhus fever appeared in the city of Philadelphia, after having entirely ceased for several successive years. In the winter of 1836–7 inflammatory pneumonia was again very prevalent, and offered more complications than it had previously done, that is the brain, and other organs, were frequently inflamed, but asthenic cases were much less frequent than they had been the previous year. In the year 1837–8, the disease became comparatively rare, and was almost exclusively confined to the spring months. The season was remarkable for its healthfulness, and pneumonia prevailed as little as other severe diseases. In the present year the disease has been rather frequent, but it is especially interesting from the number of its complications, which are, for the most part, of an acute inflammatory kind. Of these, arachnitis has been the most common and the most important.

I have alluded to the course of the disease for the last five years. It would, however, have been easy to extend my inquiries through a much longer period, if I had retained as perfect a recollection of the epidemics of previous years. It is enough for my present purpose to point out some of the varieties of pneumonia, and then we can proceed to study them in succession.

James Brown, æt. thirty-eight; entered Dec. 27, 1838; born in Ireland; in America fifteen years; labourer; intemperate. Taken ill on the 19th; was then at work on Valley Road; was in a shanty; first taken with cough and cephalalgia; had a slight pain at first in breast, not localized, and confounded by him with the general soreness. Expectoration from beginning, he thinks, as since his entrance.

The intelligence of the patient was not sufficiently developed to furnish more complete details.

The patient had been cupped on the 27th, and placed upon an expectorant mixture, consisting of the syrup of Tolu, and of senega, of each ℥iss., of tartarized antimony gr. iss.

Intense bronchial respiration at the root of the right lung posteriorly, in the extent of about three inches, with bronchophony and crepitant rhonchus extending to the axilla, and nearly to the top of the lung. The part where the bronchial respiration is most evident, is surrounded with a border of crepitant rhonchus, very fine and well characterized. Flat percussion and bronchophony corresponding with the bronchial respiration.

On the 28th, the patient was oppressed; face flushed, and of a deep red tint at each cheek; nostrils dilating at every inspiration. Cough; expectoration viscid—five or six ounces in twenty-four hours, of a rusty tint, in part consisting of the ordinary catarrhal sputa. Pulse from eighty to ninety, full; respiration thirty. Appetite diminished, but the other functions of the alimentary canal remain in the natural state.

℞. Rad. Sanguinar. Canaden. ʒiij.

Rad. Senegæ. ʒj.

M. Infuse in a pint of boiling water, and take a wine-glassful every two hours; gruel.

On the 29th, at the visit of the class, the patient was found in a most profuse perspiration, which broke out after taking three or four doses of the infusion. It had neither vomited nor purged him. Pulse softer, not less frequent. Respiration has fallen to twenty-four. Cough much more loose, and expectoration in part consists of ordinary mucus. The bronchial respiration is much less intense, and is heard at the same time with a very loose and abundant crepitus.

Suspend medicine; give the patient an infusion of flaxseed as a drink; gruel.

On the 30th, the cough mixture already directed on the 27th, was resumed. The sweating continued.

*Dec.* 31.—Much less oppressed than previously; face scarcely flushed; sweat very profuse, continuing since the 29th; less exhausted. Three or four stools only, daily; no nausea; tongue red and smooth; at tip thick; whitish-yellow fur posteriorly; cough loose, mucous; abundant expectoration—in part being muco-purulent masses, and in part thin and transparent; ʒvj. in twenty-four hours; no pain; decubitus dorsal; no expression of anxiety; appetite good; pulse seventy-six, regular, soft; respiration twenty, regular; intellect clear; percussion dull; respiration on the right side posteriorly vesicular, but a little rude at summit; still abundant crepitus in the space of three inches right below the summit, where the respiration is still a little bronchial; inferiorly there is mucous and sub-crepitant rhonchus, as well as at the axilla and along the anterior portion. Percussion flat only where the bronchial respiration remains; on the left side throughout, mucous and sonorous rhonchus. Flaxseed tea; gruel.

*January* 1*st.*—No prescription; sweating continued; a little appetite; cough very slight; expectoration rather less.

2*d.*—Expectoration mucous, in part a little purulent, with a portion still of a rusty tint. ʒii. in twenty-four hours; cough much less; face of good colour, a little florid; not flushed; no distension of nostrils; lips of good colour; sweating less abundant, but has continued since the last date without intermission. Sputa red, a little dry at edges, nearly clear. Pulse seventy-six, soft and regular; respiration twenty, regular, equable. Skin moderately warm.

Percussion anteriorly dull under right clavicle, and extending throughout the whole of the right side; but only obviously dull at summit and at base; posteriorly dull at right side, flat at upper two-thirds; sonorous at left.

Respiration posteriorly right, at upper third nearly bronchial, but vesicular sound heard. At root very bronchial, with crepitant rhonchus after coughing; at the lower third respiration is vesicular and rude with sub-crepitant rhonchus; at left side, respiration vesicular and pure although at the right, anteriorly, it is still rude. Crepitus in right axilla, posteriorly. Flaxseed tea.

3*d.* Full convalescence; still sweating, but rather less. Respiration now vesicular, but a little rude at posterior part of right lung, with sub-crepitus and crepitus in posterior margin of axilla; appetite improving; sputa moist, whitish. Oil ʒj.; flaxseed tea continued.

Discharged January 19th, 1839. The bronchial respiration had then completely disappeared.

We have begun with the disease in its most simple forms, nearly without complication,—and afterwards we shall pass to those cases which are much more difficult. This patient, as you observe, presents no symptoms connected with the brain or the abdomen; and you may perceive, by writing them in a regular series upon the black-board, we can strike out all the columns which do not refer to the functions of the respiration and the circulation. That is, the patient does not suffer more from cephalalgia, or disorder of the digestive functions, than necessarily arises from the slight degree of fever which is inseparable from all inflammatory febrile disorders; there is nothing specific or important in these symptoms. When, on the other hand, you turn to the columns which are appropriated to the symptoms connected with the circulation, you find that the pulse is frequent, and the skin warm; in other words, the patient has fever.

The symptoms of the circulation are not, however, characteristic—they may belong to a variety of other inflammatory diseases; we must seek elsewhere for the characteristic signs of pneumonia. These you will find under the head of the respiration. In the first place, the act of respiration is performed with difficulty—the nostrils dilate during each inspiration, as the muscles of respiration which are seated in the chest no longer suffice to the due performance of this function, and the muscles of the face are called in to assist. If you examine the face carefully, you may detect other symptoms connected with the respiration; the cheeks present a deep purple flush, owing to the imperfect arterialization of the blood in passing through the lungs—and the whole countenance offers that peculiar, anxious expression, which arises only from difficult respiration.

The number of inspirations is frequent—less so in the present case than in most others, even of simple pneumonia; generally they vary from twenty to fifty in the minute. Most frequently there are about thirty inspirations in the minute, when a single lung is affected in the adult. The frequency of the respiration is not only an excellent sign of pneumonia, but of the extent of the disease, for you will remark that when the brain of the patient is not enfeebled by disease or old age, the frequency of the respiration increases just in proportion to the extent of lung which is inflamed. In children the sign is even more valuable, for the respiration in them is immediately increased in frequency, when inflammation of the lungs occurs,—and from the disease frequently extending itself to both lungs, the pneumonia is often vastly more severe than in adults, and increases the frequency of the respiration sometimes to a hundred or more in the minute, but very frequently to sixty and seventy. You will often find a difficulty in ascertaining the frequency of the respiration,—if you place your hand upon the chest of the patient, you will excite his attention, and thus disturb the regularity of the respiration. The best plan is to feel the pulse of the patient, and while you keep your hand upon his wrist, you can readily count the inspirations. I lay this stress upon the frequency of the respiration, as it is a sign which is less easily ascertained than you might at first sight suppose, and one that is of great utility in the diagnosis of pneumonia. The frequency of respiration in the case which I have detailed to you is not extremely great, much less than in most cases of pneumonia. Still it is a sufficiently decided symptom to indicate with tolerable certainty a disease of the thoracic organs.

The cough in this patient is slight and rare—such is the case in pneumonia, as a general rule; at times it is even totally absent;—the symptom is therefore of little value, except as a negative sign, for the occurrence of dyspnœa in an acute disease without much cough, enables us to exclude from the calculation of probabilities all ordinary cases of bronchitis. We then seek for the seat of the disease, and usually discover it to be an acute inflammation of the serous covering of the lung, or of its parenchyma.

The expectoration was characteristic, at the entrance of the patient. By this term we mean sputa, of a viscid, tenacious consistence, transparent, and at times of a reddish, rusty tint. You will rarely find that this expectoration is altogether wanting in cases of pneumonia,—but you will very often observe that it is combined with the ordinary mucous secretion produced by bronchitis, complicating the inflammation of the substance of the lung. In this case, from the moment that resolution of the pneumonia commenced, the sputa became muco-purulent, and ceased to differ from those of the second stage of catarrh. Although I am not demonstrating a case of pneumonia of great severity, you are still able to detect the characteristic sputa, which are very rarely absent throughout the whole course of the disease. But they are not easily distinguished from those of other diseases of the lungs, excepting in the second stage. I regard the existence of these viscid and reddish sputa, as pathognomonic of pneumonia. When the local and functional signs of the disease are not recognised, you may still regard the rusty, viscid sputa, as conclusive proof of the disease. I am aware that certain rare conditions of the bronchial mucous membrane exist, in which the secretions resemble those of pneumonia; but these are so rare, that you may safely omit them in your diagnosis.

I have now pointed out to you the ordinary signs of pneumonia. You must have already satisfied yourselves that they are more or less uncertain, and that the disease cannot be recognised in a large proportion of cases by their aid. Neither will they make known the extent and exact seat of the affection, nor its rapidity of progress. We shall, in our next lecture, inquire into the degree of importance to be attached to the physical signs.

*Note.*—These lectures on pneumonia are not published precisely in the mode in which they were delivered,—a slight change, in this respect, will connect them in a natural succession, and perhaps render them more useful.

